# Management of brain metastasis with magnetic resonance imaging and stereotactic irradiation attenuated benefits of prophylactic cranial irradiation in patients with limited-stage small cell lung cancer

**DOI:** 10.1186/s12885-015-1593-2

**Published:** 2015-08-15

**Authors:** Yuichi Ozawa, Minako Omae, Masato Fujii, Takashi Matsui, Masato Kato, Shinya Sagisaka, Kazuhiro Asada, Masato Karayama, Toshihiro Shirai, Kazumasa Yasuda, Yutaro Nakamura, Naoki Inui, Kazunari Yamada, Koshi Yokomura, Takafumi Suda

**Affiliations:** 1 Department of Respiratory Medicine, Respiratory Disease Center, 3453 Mikatahara, Kita-ku, Hamamatsu, Shizuoka 433-8558 Japan; 2 Department of Radiation Oncology, Seirei Mikatahara General Hospital, 3453 Mikatahara, Kita-ku, Hamamatsu, Shizuoka 433-8558 Japan; 3 Department of Respiratory Medicine, Shizuoka General Hospital, 4-27-1 Kita-Ando, Aoi-ku, Shizuoka, Shizuoka 420-8527 Japan; 4 Department of Respiratory Medicine, Iwata City Hospital, 512-3 Okubo, Iwata, Shizuoka 438-0002 Japan; 5 Department of Clinical Oncology, 1-20-1 Handayama, Higashi Ward, Hamamatsu, Shizuoka 431-3192 Japan; 6 Department of Clinical Pharmacology and Therapeutics, 1-20-1 Handayama, Higashi Ward, Hamamatsu, Shizuoka 431-3192 Japan; 7 Second Division, Department of Internal Medicine, Hamamatsu University School of Medicine, 1-20-1 Handayama, Higashi Ward, Hamamatsu, Shizuoka 431-3192 Japan

**Keywords:** Small cell lung cancer, Prophylactic cranial irradiation, Stereotactic irradiation, Magnetic resonance imaging, Brain metastasis

## Abstract

**Background:**

Magnetic resonance imaging (MRI) enables a more sensitive detection of brain metastasis and stereotactic irradiation (SRI) efficiently controls brain metastasis. In limited-stage small cell lung cancer (LS-SCLC), prophylactic cranial irradiation (PCI) in patients with good responses to initial treatment is recommended based on the survival benefit shown in previous clinical trials. However, none of these trials evaluated PCI effects using the management of brain metastasis with MRI or SRI. This study aimed to determine the effects of MRI and SRI on the benefits of PCI in patients with LS-SCLC.

**Methods:**

The clinical records of pathologically proven SCLC from January 2006 to June 2013 in facilities equipped with or had access to SRI in Japan were retrospectively reviewed. Patients with LS-SCLC and complete or good partial responses after initial treatment were included in the study and analyzed by the Kaplan-Meier method.

**Results:**

Of 418 patients with SCLC, 124 met criteria and were divided into patients receiving PCI (PCI group; *n* = 29) and those without PCI (non-PCI groups; *n* = 95). At baseline, ratios of patients with stage III were significantly advantageous for the non-PCI group, although younger age and high ratios of complete response and MRI confirmed absence of brain metastasis were advantageous for the PCI group. Neither median survival times (25 *vs.* 34 months; *p* = 0.256) nor cumulative incidence of brain metastasis during 2 years (45.5 *vs.* 30.8 %; *p* = 0.313) significantly differed between the two groups. Moreover, these factors did not significantly differ among patients with stage III disease (25 *vs.* 26 months; *p* = 0.680, 42.3 *vs.* 52.3 %; *p* = 0.458, respectively).

**Conclusion:**

PCI may be less beneficial in patients with LS-SCLC if the management with MRI and SRI is available.

## Background

Small cell lung cancer (SCLC) comprises approximately 15 % of all lung cancers, and usually progresses rapidly and preferentially metastasizes to the brain. Even with early detection before distant metastasis and curative treatments, 50–60 % of patients with SCLC develop brain metastasis (BM) within 2 years [1–4]. Because the presence of BM indicates poor prognosis, patients with SCLC and symptomatic or asymptomatic BM have median survival times (MSTs) of only 4–8 months even under systemic treatment [5].

Several clinical trials have evaluated the efficacy of prophylactic cranial irradiation (PCI), and most have shown significant reductions in the occurrence of BM and survival improvement in patients with limited-stage SCLC (LS-SCLC) and good responses to initial treatment [1–4]. However, to our knowledge, only two of the 17 reported trials required confirmation of the absence of BM before PCI with contrast-enhanced computed tomography (CT) [6, 7], and none of them used magnetic resonance imaging (MRI) for detecting BM [1–4, 6–20].

Seute et al. revealed that BM was detected in 10 % of patients with SCLC during the CT era and in 24 % during the MRI era, and the adaptation of MRI decreased the frequency of PCI from 42 to 13 % [21]. Moreover, Manapov et al. reported that cranial MRI immediately before PCI detected BM in 32.5 % of patients with LS-SCLC who had been assessed with complete response (CR) [22]. Thus, a strict selection of patients receiving PCI, which excludes those who have BM after the initial treatment using cranial MRI, may affect BM occurrence, survival times, and PCI benefits. However, no studies evaluated PCI effects in such a cohort.

SRI is recently reported to be capable of controlling single or multiple BM, at least locally, among patients with cancer, including SCLC [23–25], and it is extensively used in clinical practice in Japan, although the improvement of survival time by SRI remains unknown. Harris et al. reported that SRI efficiently controlled BM in patients with a poor prognosis of SCLC who developed BM after PCI or whole brain radiotherapy (WBRT) [24] The MST of 5.9 months indicated that administering SRI to patients with BM could prolong survival time. Although more evidence is required, SRI could also affect the importance of PCI.

We hypothesized that precise patient selection without BM using cranial MRI immediately before PCI and efficient local control of BM with SRI may limit previously reported benefits of PCI. In the present study, we retrospectively compared the incidences of BM and survival time between PCI-treated and -untreated patients in facilities with access to MRI and SRI.

## Methods

Data from patients with pathologically proven SCLC were collected from January 2006 to June 2013 at the Hamamatsu University School of Medicine, Seirei Mikatahara General Hospital, Shizuoka General Hospital, and Iwata City Hospital. All four participating facilities were cancer-designated hospitals in Japan and were equipped with or had access to SRI and MRI. Medical records were reviewed, and age, sex, smoking history, laboratory findings, type of and response to initial treatment, treatment for BM, and outcomes were analyzed. The study was approved by the Institutional Review Board of Hamamatsu University School of Medicine, Seirei Mikatahara General Hospital, Iwata City Hospital, and Shizuoka General Hospital.

Of 418 newly diagnosed patients with SCLC during this period, 124 patients with LS-SCLC with CR or good partial response (gPR) to initial treatment were enrolled in this study. Disease stages were determined based on the initial staging investigations, including chest and abdomen CT, bone screening by whole-body 18F-fluorodeoxyglucose positron emission tomography or scintigraphy, and brain screening using contrast-enhanced CT or MRI. Limited-stage was defined as limited disease originating from the hemithorax that may include the mediastinum or supraclavicular lymph nodes. Malignant pleural or pericardial effusions and contralateral supraclavicular lymph nodes were excluded.

Patients who developed BM during treatment were preferentially treated with SRI following discussions with radiation oncologists. The response to initial treatment was determined by imaging tests requested by the treating doctors and were interpreted by the reporting radiologist. According to RECIST version 1.1, CR was characterized by the disappearance of all target and non-target lesions and the reduction of short axes of all lymph nodes to <10 mm with tumor marker levels at less than the reference range. Similarly, gPR was defined as the disappearance of lesions, but with elevated or unknown tumor marker levels and 10–15-mm short axes of lymph nodes. Chest CT was used to distinguish between CR and gPR. Although cranial scans after initial treatment were unnecessary, contrast-enhanced MRI was preferentially used for confirming the absence of BM.

Overall survival was defined as the interval between the date of pathological diagnosis and that of the final follow-up visit or death, and survival time after BM detection was defined as the interval between the date of BM detection with any imaging modality and that of the final follow-up visit or death. MST and the cumulative incidence of BM were estimated by the Kaplan-Meier method [26], and groups were compared using the log-rank test. Categorical data were compared between groups using the chi-square test for independence, and continuous data were compared using Student’s t- test [27]. All tests were two-sided, and a *p*-value of <0.05 was considered statistically significant. All statistical analyses were performed using the PASW Statistics version 18.0 for Windows (SPSS Inc., Chicago, USA).

## Results

After enrollment, 124 patients were divided into PCI-treated (PCI group; *n* = 29) and untreated (non-PCI group; *n* = 95) patients. At baseline, the ratio of clinical stage III patients was significantly higher in the PCI group than the non-PCI (82.8 *vs.* 51.6 %; *p* = 0.0009), which would be advantageous for the latter group, although most other significant or non-significant differences between the two groups, including younger age (65 *vs.* 74; *p* < 0.0001) and a higher ratio of CR (86.2 *vs.* 60.0 %; *p* = 0.0052), were advantageous for the PCI group (Table 1). On cranial MRI immediately before PCI, the absence of BM was confirmed in 28 patients of the PCI group (96.5 %) and in 57 patients of the non-PCI group (60.0 %), presenting a significant difference (*p* < 0.0001) and an advantage for the PCI group. No cranial CT scans were performed.

**Table 1:** Patient characteristics

	All (*n* = 124)	PCI (*n* = 29)	non-PCI (*n* = 95)	*P*
Age, year (range)	72 (37–88)	65 (37–74)	74 (37–88)	<0.0001
Male	113 (91)	27 (93)	86 (90)	0.778
Smoking status				0.721
Current	61 (49)	15 (52)	46 (48)	
Former	60 (48)	14 (48)	46 (48)	
Never	2 (2)	0	2 (2)	
Unknown	1 (1)	0	1 (1)	
Number of packets	46 (0–210)	60 (12–140)	45 (0–210)	0.226
Clinical stage				0.0009
Stage I/II	51 (41)	5 (17)	46 (49)	
Stage III	73 (59)	24 (83)	49 (52)	
ECOG PS				0.337
0	70 (56)	20 (69)	50 (53)	
1	50 (40)	8 (28)	42 (44)	
2	4 (3)	1 (3)	3 (3)	
Initial treatment				0.094
Chemoradiotherapy	61 (49)	19 (66)	42 (44)	
Radiotherapy	4 (3)	0 (0)	4 (4)	
Surgery	41 (34)	8 (28)	33 (35)	
Chemotherapy	18 (15)	2 (7)	16 (17)	
Response to initial treatment				0.0052
Complete response	82 (66)	25 (86)	57 (60)	
Good partial response	42 (34)	4 (14)	38 (40)	
Cranial MRI before PCI	85 (69)	28 (97)	57 (60)	<0.0001
Observation period (month)	20 (6–94)	20 (6–82)	20 (6–94)	0.945

Data are numbers of patients (percentage of patients) otherwise indicated

*PCI* prophylactic cranial irradiation; *ECOG PS* Eastern Cooperative Oncology Group performance status

**Fig. 1:**
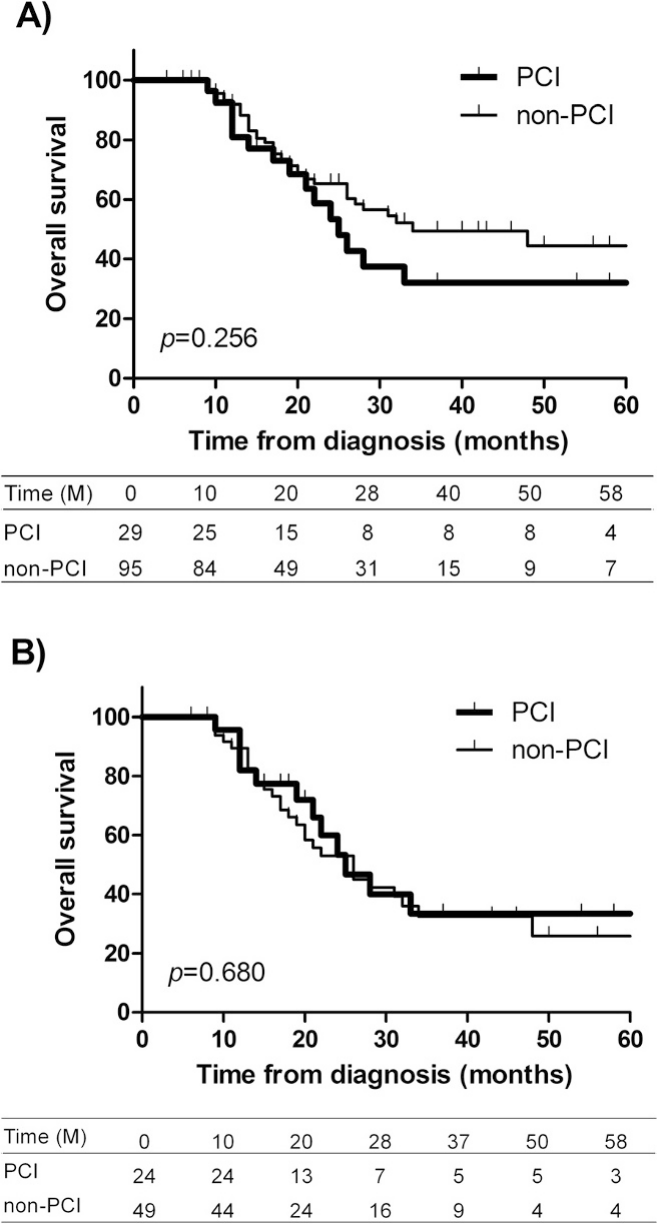
Prophylactic cranial irradiation (PCI) and overall survival of all patients (**a**) and of patients with stage III disease (**b**), M: months

**Fig. 2:**
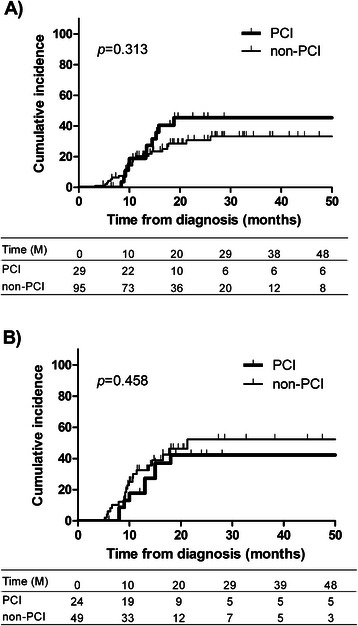
Prophylactic cranial irradiation (PCI) and cumulative incidence of brain metastases among all enrolled patients (**a**) and in those with stage III disease (**b**), M: months

Survival curves after the diagnosis of SCLC in 124 enrolled patients are shown in Fig. 1a. MSTs were 25 and 34 months in the PCI and non-PCI groups, revealing no significant difference between the groups (*p* = 0.256). To correct for differences in the disease stage, we only analyzed patients with stage III disease (Fig. 1b) and revealed similar survival curves and MSTs (25 *vs.* 26 months, respectively, *p* = 0.680). Curves for the cumulative occurrence of BM are shown in Fig. 2a and b. Among the 124 enrolled patients, BM developed at 2 years from diagnosis in 45.5 and 30.8 % in the PCI and non-PCI groups, respectively (*p* = 0.313). Similarly, among patients only with stage III disease, occurrence rates during 2 years were 42.3 and 52.3 % in the PCI and non-PCI groups, respectively, and did not significantly differ between the two groups (*p* = 0.458). Furthermore, we analyzed patients confirmed to not have BM with cranial MRI after initial treatment. The BM occurrence rates during 2 years were 43.0 and 38.4 % in the PCI and non-PCI groups, respectively (*p* = 0.865).

During the observation period, 36 patients (29.0 %) developed BM: 11 in the PCI group (37.9 %) and 25 in the non-PCI group (26.3 %). Among them, 25 were asymptomatic and 17 had only one intracranial lesion at the initial detection of BM. Subsequently, 7 and 18 patients in the PCI (63.6 %) and non-PCI (72.0 %) groups received SRI therapy. Of these 25 patients, 11, 8, and 6 received γ-knife, X-knife, and stereotactic radiotherapy, respectively. All seven patients in the PCI group received SRI only once. In contrast, six, three, and one patients received two, three, and five separate sessions, respectively, of salvage SRI therapy in the non-PCI group. WBRT was administered as salvage therapy in 13 patients (41.7 %), 11 of which belonged to the non-PCI group. Two patients in the PCI group received WBRT for short-term palliation. No patient underwent craniotomy.

## Discussion

In the present study, we retrospectively evaluated the efficacy of PCI in patients with LS-SCLC, some of whom received BM management with SRI and cranial MRI. There was no significant difference in BM occurrence or survival between patients with and without PCI.

PCI in patients with good responses to initial treatment is highly recommended based on previous clinical trials. In contrast, the present study revealed that patients with PCI did not benefit from BM management. There are some differences between previous studies and the present study. At first, high incidence of BM examination using cranial MRI was observed: 96.5 and 60.0 % in the PCI and non-PCI groups, respectively. On the other hand, all but two trials did not require confirmation of the absence of BM before PCI [1–4, 6–20], which raised the possibility that these previous trials contained more patients actually having BM at the time of PCI. Because BM screening confirmed that patients were free from BM and that patients without BM may represent a better prognostic population [22], the cranial MRI after initial treatment would be one of the primary causes. In the present study, the cumulative incidence of BM in patients who did not receive PCI was only 30.8 %, which is lower than that previously reported (50–60 %).

Although there is no available data about the efficacy of PCI with the adaptation of SRI, reports indicating the benefit of SRI in patients with SCLC have been increasing. Harris et al. reported that patients with SCLC who developed BM after PCI or WBRT and were treated by γ-knife had an MST of 5.9 months despite poor prognostic backgrounds [24]. Wegner et al. reported that BM occurrences after PCI in patients with SCLC were efficiently salvaged with SRI, and the MST in those SRI-treated patients was 9 months [28]. Survival benefit, at least partly owing to SRI, can lower the contribution of PCI on survival, which may explain the results of the present study. Thus, although PCI remains strongly recommended based on previously reported benefits, these benefits may be attenuated if the management with MRI and SRI is available

Neurologic toxicities of PCI were not assessed in the present study. The association between neurological toxicity and PCI has been debatable since the past 30 years [29–31]. The PCI strategy involving irradiation of brains in patients with LS-SCLC for treating undetectably small BM has reasonably provided a 50–60 % incidence of BM after the initial treatment. However, recent prospective studies, including two trials for PCI [32, 33] and two for WBRT [33, 34], have shown substantial cognitive impairments after whole brain irradiation. Gondi et al. demonstrated an association between PCI and declines in Hopkins Verbal Learning Test and self-reported cognitive function between 410 patients with PCI and 173 control patients who did not develop BM [32]. Uno et al. reported that only 12 of the 139 patients with LS-SCLC (8.6 %) received PCI in Japan [35], possibly because of concerns about the neurocognitive impairment. Although it is generally considered that the benefits of PCI should be favorably weighed against the associated adverse effects, we need to reconsider the benefits of PCI based on the understanding of exact adverse effects.

There are limitations to the present study. First, the sample size was small, which may not particularly be sufficient for analyzing the survival time because of considerably lesser expectancy of risk reduction, 14–18 % in survival time, while 50–68 % is reported in the BM prevention [1–4, 36]. Second, the number of patients in this retrospective study was unequal in the two groups. Furthermore, the limited number in the PCI group could affect the statistical power for detecting the benefits of PCI. A higher ratio of early clinical stage in the non-PCI group would be advantageous for the non-PCI group, and younger age, higher ratios of CR and MRI confirmed absences of BM were advantageous for the PCI group, which may limit the current results. However, it is not realistic to interventionally and prospectively verify the benefit of PCI at present, and we believe our study can offer the opportunity to reconsider the adaptation of PCI. Additional analysis with patients with stage III disease reproduced similar survival and BM occurrence curves in those with and without PCI. The absence of clear criteria for the adaptation of PCI, cranial MRI, and SRI, which were individually decided by the physicians, is another major limitation. Considering these limitations, we must recognize that the results of our retrospective study are not practice-changing and further studies are needed to confirm our results.

## Conclusion

This retrospective study suggests that treatments with PCI did not relate the benefit in BM occurrence or survival in patients for whom SRI and MRI were available. Thus, in the situation that the adaptation of SRI and MRI are available for the management of BM, PCI may be less beneficial than previously reported. Based on the current results, a further prospective, observational study is in progress.

## Abbreviations

MRI: Magnetic resonance imaging
SRI: Stereotactic irradiation
PCI: Prophylactic cranial irradiation
LS: Limited stage
SCLC: Small cell lung cancer
BM: Brain metastasis
MST: Median survival time
CT: Computed tomography
WBRT: Whole brain radiation therapy
CR: Complete responses
gPR: Good partial responses
